# Virulence Factors in *Staphylococcus* Associated with Small Ruminant Mastitis: Biofilm Production and Antimicrobial Resistance Genes

**DOI:** 10.3390/antibiotics10060633

**Published:** 2021-05-25

**Authors:** Nara Cavalcanti Andrade, Marta Laranjo, Mateus Matiuzzi Costa, Maria Cristina Queiroga

**Affiliations:** 1 MED–Mediterranean Institute for Agriculture, Environment and Development, Instituto de Investigação e Formação Avançada, Universidade de Évora, Pólo da Mitra, Ap. 94, 7006-554 Évora, Portugal; naraegabriel@hotmail.com (N.C.A.); mlaranjo@uevora.pt (M.L.); 2 Federal University of the São Francisco Valley, BR 407 Highway, Nilo Coelho Irrigation Project, s/n C1, Petrolina 56300-000, PE, Brazil; mateus.costa@univasf.edu.br; 3 Departamento de Medicina Veterinária, Escola de Ciências e Tecnologia, Universidade de Évora, Pólo da Mitra, Ap. 94, 7006-554 Évora, Portugal

**Keywords:** mastitis, staphylococci, virulence factors, genes, biofilm, antimicrobial resistance

## Abstract

Small ruminant mastitis is a serious problem, mainly caused by *Staphylococcus* spp. Different virulence factors affect mastitis pathogenesis. The aim of this study was to investigate virulence factors genes for biofilm production and antimicrobial resistance to β-lactams and tetracyclines in 137 staphylococcal isolates from goats (86) and sheep (51). The presence of *coa*, *nuc*, *bap*, *icaA*, *icaD*, *blaZ*, *mecA*, *mecC*, *tetK*, and *tetM* genes was investigated. The *nuc* gene was detected in all *S. aureus* isolates and in some coagulase-negative staphylococci (CNS). None of the *S. aureus* isolates carried the *bap* gene, while 8 out of 18 CNS harbored this gene. The *icaA* gene was detected in *S. aureus* and *S. warneri*, while *icaD* only in *S. aureus*. None of the isolates carrying the *bap* gene harbored the *ica* genes. None of the biofilm-associated genes were detected in 14 isolates (six *S. aureus* and eight CNS). An association was found between *Staphylococcus* species and resistance to some antibiotics and between antimicrobial resistance and animal species. Nine penicillin-susceptible isolates exhibited the *blaZ* gene, questioning the reliability of susceptibility testing. Most *S. aureus* isolates were susceptible to tetracycline, and no cefazolin or gentamycin resistance was detected. These should replace other currently used antimicrobials.

## 1. Introduction

Mastitis is the inflammation of the mammary gland, mainly due to intramammary infection (IMI). In small ruminants, this disease is considered a serious economic issue due to the mortality of lactating females, cost of treatment, reduced milk yield and quality [1,2], as well as a public health concern associated with risk of consumer food poisoning [3,4]. Several pathogens can cause mastitis in small ruminants; however, species of staphylococci are the most frequently isolated microorganisms from goat and sheep milk [2,5,6,7,8].

*Staphylococcus aureus* is one of the main pathogens associated with mastitis in small ruminants [9]. Incidence of clinical mastitis in sheep due to this bacterium may reach 20% with a mortality rate between 25% and 50%, and the affected mammary halves in surviving animals are frequently destroyed. Chronic mastitis may cause a 25 to 30% reduction in milk yield from the affected udder [10].

Coagulase negative staphylococci (CNS), although not as virulent as *S. aureus*, often cause subclinical mastitis in small ruminants [5,11,12,13]. This type of infection, most times not detected by the farmer, clearly reduces milk production, also changing milk composition, indirectly impairing the milk product’s properties [14]. CNS are the most prevalent pathogens of the mammary gland in goats and sheep with subclinical mastitis, affecting 60% to 80.7% in goats and 45% to 48% in sheep [1]. Other authors have reported as much as 70.1% of subclinical mastitis in sheep is caused by CNS [5].

Virulence factors are bacterial molecules that enhance their capacity to establish and to survive within the host and, thus, contribute to bring damage to the host. Staphylococci possess a wide array of virulence factors [15].

Coagulase enzyme acts on plasma fibrinogen to form fibrin clots that protect the microorganisms from phagocytosis and shelter them from other cellular and soluble host defence mechanisms. This enzyme, encoded by the *coa* gene, is commonly used to distinguish coagulase positive staphylococci (CPS), namely *S. aureus*, *S. intermedius*, and *S. pseudintermedius*, from CNS species [16]. Nevertheless, this gene has been found also in species known as CNS such as *S. epidermidis*, *S. chromogenes*, and *S. hominis* [17]. The *coa* gene has also recently been associated with biofilm production [18].

The staphylococcal nuclease is a thermostable nuclease encoded by the *nuc* gene [19], which hydrolyzes DNA and RNA in host cells, causing tissue destruction and spreading of staphylococci [20], also promoting the escape of microorganisms when retained by neutrophil extracellular traps (NETs), allowing the bacteria to evade this host defence mechanism [21,22]. For decades, the *nuc* gene has been considered the golden standard for *Staphylococcus aureus* identification and is still used presently [23,24,25]. However, the *nuc* gene has been detected in staphylococci of animal origin other than *S. aureus* [26]. Moreover, the *nuc* encoded staphylococcal thermonuclease is a biofilm inhibitor that degrades the environmental DNA (eDNA) associated with biofilm [27,28].

The production of biofilm is considered a major virulence factor that, besides protecting from host defence mechanisms, also shields bacteria against antimicrobial agents [29]. Furthermore, the persistence of biofilm-producing isolates in the dairy environment enhances the dispersal of virulence factors though the transfer of genetic material to other bacteria [30]. Biofilm major components are an exopolysaccharide matrix, proteins, and eDNA, along with the bacterial cells [31]. The exopolysaccharide, polysaccharide intercellular adhesin (PIA), is also a non-protein adhesin [32] assisting in bacterial adhesion to different surfaces, comprising the first critical event in the establishment of an infection [33]. Staphylococcal PIA is encoded by the *ica* operon [34], and biofilm-associated protein (Bap) is a surface protein connected to the cell wall encoded by the *bap* gene [35].

Antimicrobial resistance (AMR) is a major problem hampering the treatment of an ever increasing range of infections caused by bacteria [36]. Staphylococci resistance has been reported for different antimicrobials used for mastitis control in small ruminants [7,36,37,38]. Genes often described in *Staphylococcus* spp. isolated from the milk of small ruminants are *bla*Z and *mec*A, responsible for β-lactam resistance and *tet*K and *tet*M, accounting for tetracycline resistance [39,40,41]. The presence of resistant bacteria in contaminated food products may lead to the transfer of resistance genes to the indigenous microbiota in the human gut [42].

The aim of this study was to identify *Staphylococcus* species isolated from small ruminants’ milk samples and investigate the presence of genes encoding virulence factors associated with both biofilm (*coa*, *nuc*, *bap*, *icaA*, and *icaD*) and antimicrobial resistance to β-lactams (*blaZ*, *mecA*, and *mecC*) and tetracyclines (*tetK* and *tetM*).

## 2. Results and Discussion

### 2.1. Bacteriological Results

From the 646 milk samples collected from goats (508) and sheep (138), bacteriological cultures resulted positive in 191 samples: 131 goat milk and 60 sheep milk. A total of 137 staphylococcal isolates were recovered, of which 86 were isolated from goat and 51 from sheep milk samples.

### 2.2. Staphylococci Identification

Excellent (96 to 99% probability) and very good (93 to 95% probability) identification was observed for most *Staphylococcus*. Unidentified isolates and isolates with low discrimination results were confirmed by 16S rRNA gene sequencing.

Concerning goat milk samples, four *S. aureus*, one *Staphylococcus* sp., and 12 different CNS species were found: *S. caprae* (25), *S. chromogenes* (10), *S. epidermidis* (11), *S. simulans* (8), *S. warneri* (7), *S. capitis* (4), *S. lentus* (4), *S. hominis* (4), *S. hyicus* (3), *S. auricularis* (2), *S. haemolyticus* (2), and *S. equorum* (1).

On the other hand, 31 *S. aureus* and seven different CNS species were recovered from sheep milk samples: *S. chromogenes* (9), *S. epidermidis* (3), *S. auricularis* (2), *S. haemolyticus* (2), *S. simulans* (2), *S. lentus* (1), and *S. rostri* (1). *Staphylococcus rostri* has only been seldom isolated from the milk of a sheep with subclinical mastitis [43,44].

In the CNS group, *S. caprae* was the most found species and was isolated only from goat’s milk samples. It is a commensal organism that prevails in the skin of the goat udder [19] This species is most commonly found in cases of goat mastitis [37,45,46,47], but it was also isolated from sheep [5,48], buffalo [17], and cow’s milk [49].

In this study, other *Staphylococcus* species were only isolated from goats: *S. warneri*, *S. capitis*, *S. hominis*, *S hyicus*, and *S. equorum*. This was probably because the sheep sampling was smaller, since all these species have been isolated before from sheep milk by several other authors [44].

### 2.3. Biofilm Production

Of the 137 *Staphylococcus* isolates analyzed, 103 were biofilm producers (75%). Biofilm-forming isolates belong to the following species: *S.* *aureus* (29/35), *S.* *caprae* (22/25), *S.* *chromogenes* (12/19), *S. epidermidis* (11/14), *S.* *warneri* (7/7), *S.* *simulans* (6/10), *S.* *auricularis* (4/4), *S.* *capitis* (3/4), *S. lentus* (3/5), *S. haemolyticus* (2/4), *S. hominis* (2/4), *S. equorum* (1/1), and *Staphylococcus* sp. (1/1). All *S. epidermidis* goat isolates were found to produce biofilm in the present study, in accordance with the findings of others authors that reported *S. epidermidis* as the most commonly found species in biofilm-associated human infections [50]. However, none of the sheep *S. epidermidis* isolates were biofilm producers. In fact, other studies had already reported only 8% of biofilm-producing isolates among sheep mastitis *S. epidermidis* [51].

### 2.4. Genes Associated to Biofilm

We investigated the presence of *coa* and *nuc* genes in all 137 staphylococcal isolates, mainly for identification purposes and due to historical reasons. In fact, the ability of a strain to produce coagulase, encoded by the *coa* gene, is the basis of the primary classification of staphylococci in coagulase-positive or coagulase-negative [16].

**Figure 1:**
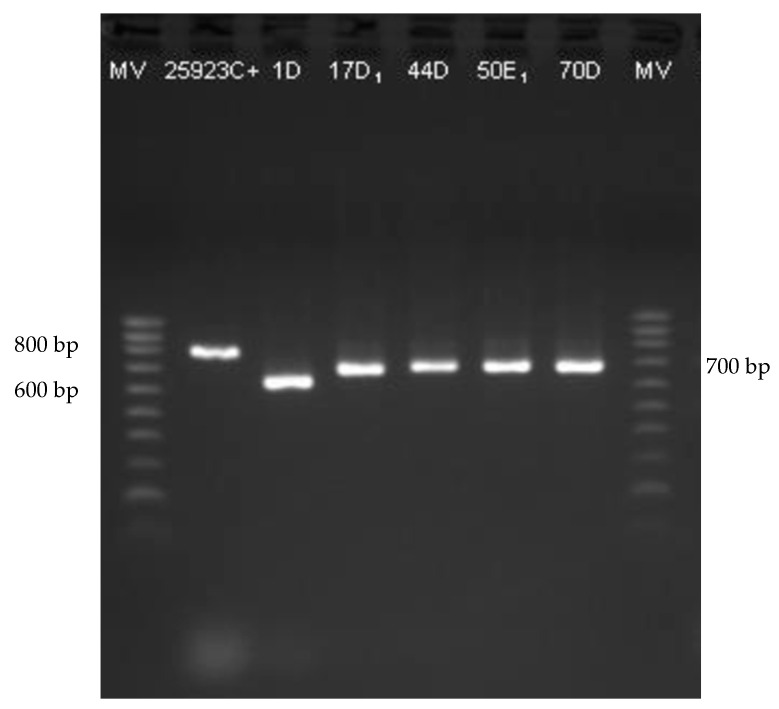
Agarose gel electrophoresis of *S. aureus coa* gene PCR products. NZYDNA Ladder V (200–1000 bp) (NZYTech, Lisbon, Portugal).

All *S. aureus* isolates (35) harbored the *coa* gene, as well as isolate B200E1, not identified to the species level. Based on this result, this isolate was probable also *S. aureus*. Therefore, the 101 *Staphylococcus* isolates not carrying the *coa* gene were confirmed as CNS. Furthermore, in the present study, different amplicons of the *coa* gene with band sizes ranging from 400 to 900 bp were detected (Figure 1), as already reported by others [52,53,54,55]. In fact, the *coa* gene also has a discriminatory power between isolates because of the heterogeneity of its 3’ variable region containing 81-bp tandem short sequence repeats (SSR) [56,57,58].

The *nuc* gene was detected in 67 out of 137 isolates (48.9%), of which only 35 were *S.* *aureus*. The other *nuc* positive isolates included: *S. chromogenes* (8), *S. warneri* (4), *S. auricularis* (3), *S. caprae* (3), *S. hyicus* (3), *S. lentus* (3), *S. epidermidis* (2), *S. simulans* (2), *S. capitis* (1), *S. haemolyticus* (1), *S. hominis* (1), and *Staphylococcus* sp. (1). Furthermore, an association was found between the *Staphylococcus* species and the presence of the *nuc* gene (χ^2^ = 70.968, df = 14, *p* < 0.001). In fact, all *S. aureus* harbor the *nuc* gene, while most CNS (70/101) do not. However, the *nuc* gene was also detected in more than 50% of the isolates in some CNS species: *S. warneri* (4/7), *S. lentus* (3/5), *S. auricularis* (3/4), and *S. hyicus* (3/3).

The presence of the *nuc* gene was used in the past to identify *S. aureus* [23,25]. The *nuc* gene is present in most *S. aureus* isolates; however, some isolates not carrying this gene have been described [59,60]. Moreover, the *nuc* gene has also been detected in other species of *Staphylococcus*, both CPS and CNS [61,62].

For the detection of the biofilm production genes, *bap*, *icaA*, and *icaD*, the 44 *nuc*-positive biofilm-producing isolates were selected. *nuc*-positive biofilm-producing staphylococci and biofilm-associated genes are shown in Table 1.

The *bap* gene was amplified in eight isolates: *S. chromogenes* (5), *S. auricularis* (1), *S. simulans* (1), and *S. warneri* (1). None of the *S. aureus nuc*-positive biofilm-producing isolates carries the *bap* gene. In fact, the *bap* gene has been reported mainly in *S. aureus* strains isolated from cattle [24,63,64]. However, Martins et al. [65] have detected the *bap* gene in four sheep milk *S. aureus* isolates. In our study, 8 out of 18 CNS *nuc*-positive biofilm-producing isolates harbored the *bap* gene. The *bap* gene encodes a cell wall associated protein named Bap (for biofilm associated protein), which enhances biofilm formation as it mediates bacterial primary attachment to abiotic surfaces and intercellular adherence [35]. Other studies have reported the presence of the *bap* gene in several CNS isolates [66].

The presence of the *icaA* gene was detected in seven isolates: *S. aureus* (5) and *S. warneri* (2). On the other hand, the *icaD* gene was present in 19 *S. aureus* isolates. Furthermore, five *S. aureus* isolates carried both *icaA* and *icaD* genes simultaneously. Xu, Tan, Zhang, Xia, and Sun [59] detected the *icaD* gene in 20 out of 28 *S. aureus* bovine mastitis isolates, while it was not detected in any of the 76 CNS analyzed. The same authors reported the absence of the *icaA* gene in all analyzed staphylococcal isolates [59].

No isolate carrying the *bap* gene harbored the *ica* operon genes, as reported before by other authors [67]. However, Marques et al. [68] found one single bovine mastitis *S. aureus* isolate (out of 20) that simultaneously carried *bap*, *icaA*, and *icaD*.

None of the three biofilm-associated genes were detected in 14 of the *nuc*-positive biofilm-producing isolates: *S. aureus* (6) and CNS (8). Other authors have also reported the absence of *bap*, *icaA*, and *icaD* genes in biofilm-producing *S. aureus* [24,69,70]. Despite no association being found between the presence of the *nuc* gene and biofilm production, most biofilm-producing isolates harbored the *nuc* gene (53.4%), while it was only detected in about 35% of the non-producers. Nevertheless, Kiedrowski, Kavanaugh, Malone, Mootz, Voyich, Smeltzer, Bayles, and Horswill [28] described an inverse correlation between Nuc thermonuclease activity and biofilm formation and confirmed the important role for eDNA in the *S. aureus* biofilm matrix.

Apparently, CNS produce biofilm mainly via Bap, as already suggested by Zuniga et al. [71], who found the *bap* gene to be more frequently present in CNS when compared to CPS.

Meanwhile, most *S. aureus* seem to form biofilm through PIA since they harbor the *icaA* and *icaD* genes. Other authors have reported that a low prevalence of the *bap* gene in *S. aureus* indicates that the *ica* operon-dependent mechanism may be the main responsible for the adhesion and biofilm formation in this species [68]. Notwithstanding, it has been reported that biofilm synthesis in *S. aureus* can also be encoded by the *bap* gene [72].

Other biofilm formation mechanisms in staphylococci not harboring the classical biofilm-production genes, *bap*, *icaA*, and *icaD*, need to be explored. Furthermore, some of the isolates not carrying *bap*, *icaA*, and *icaD* also did not harbor the *coa* gene, which has been reported as associated with biofilm formation [18]. However, the *nuc* gene might be an important factor to consider since all 44 isolates were biofilm producers and harbored the *nuc* gene, although Nuc has been referred to as a biofilm inhibitor [27,28].

### 2.5. Antimicrobial Resistance

Out of 137 staphylococcal isolates analyzed for antimicrobial susceptibility, 15 were multidrug resistant, 36 were non-susceptible to two antimicrobial categories, and 61 to one antimicrobial category, according to the classification proposed by Magiorakos et al. [73]. Moreover, no antimicrobial resistances were detected in 24 staphylococcal isolates.

Staphylococci isolated from milk from small ruminants with mastitis are known for their multiresistance [74]. In this work, the multidrug resistant (MDR) isolates belonged to the following species: *S. aureus* (8), *S. lentus* (3), *S. chromogenes* (2), *S. caprae* (1), and *S. warneri* (1). Contrarily, Taponen and Pyorala [75] reported that multiresistance was more common in CNS than in *S. aureus* from bovine mastitis.

**Figure 2:**
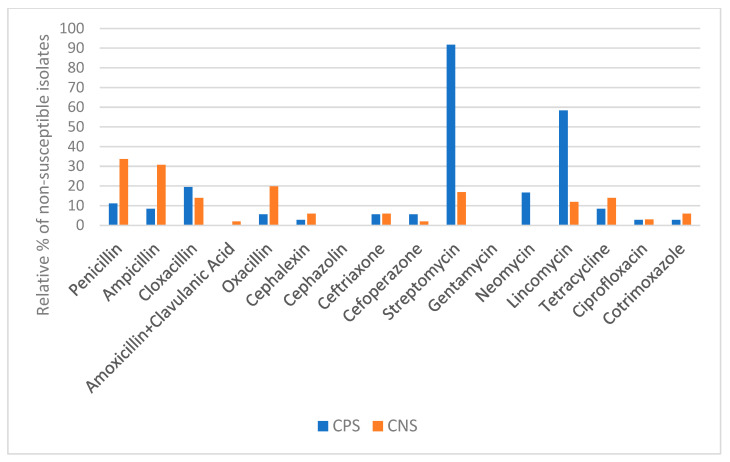
Susceptibility patterns of CPS (*n* = 36) and CNS (*n* = 101) isolates to antimicrobials.

Susceptibility patterns of CPS and CNS isolates are shown in Figure 2. For most antimicrobials tested, a higher percentage of resistant isolates was observed among CNS when compared to CPS. Vasileiou et al. [76] also reported more resistant CNS isolates than *S. aureus*. However, mastitis caused by CNS responds much better to antimicrobial treatment than *S. aureus* mastitis [75].

Staphylococcal isolates were mainly non-susceptible to streptomycin (50/137), penicillin (38/137), ampicillin (34/137), lincomycin (33/137), oxacillin (22/137), cloxacillin (21/137), and tetracycline (17/137), as previously reported [77] (Figure 2). Moreover, most CPS isolates were non-susceptible to streptomycin and lincomycin. On the other hand, CNS isolates were mostly non-susceptible to the β-lactams and tetracyclines.

**Figure 3:**
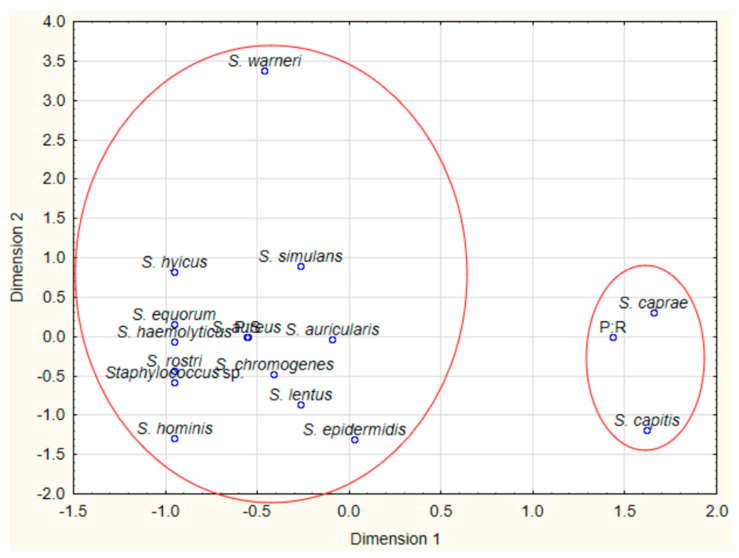
CA biplots of the relationship between bacterial species and tolerance to the antimicrobials penicillin (*P*), ampicillin (AMP), streptomycin (S), and lincomycin (MY).

In addition, an association was found between *Staphylococcus* species and antimicrobial resistance to penicillin (χ^2^ = 45.981, df = 14, *p* < 0.001), ampicillin (χ^2^ = 48.327, df = 14, *p* < 0.001), streptomycin (χ^2^ = 137.705, df = 28, *p* < 0.001), lincomycin (χ^2^ = 156.536, df = 28, *p* < 0.001), cephalexin (χ^2^ = 57.219, df = 28, *p* < 0.05), and tetracycline (χ^2^ = 51.626, df = 28, *p* < 0.05). Regarding the results shown by the correspondence analysis, most *S. caprae* and *S. capitis* isolates were resistant to penicillin and ampicillin, while all other staphylococci were mostly susceptible to these antimicrobials (Figure 3). Most *S. aureus* isolates exhibited an intermediate susceptibility pattern to streptomycin and lincomycin [78]. Additionally, all *S. hyicus* isolates were resistant to streptomycin, while *S. lentus* and *S. rostri* were resistant to lincomycin (Figure 3).

No staphylococci resistant to cefazolin and gentamycin were identified. Moreover, no non-susceptible *S. aureus* isolates were found to amoxicillin + clavulanic acid. A number of CNS isolates, although resistant to penicillinase-labile penicillins, were susceptible to amoxicillin + clavulanic acid, which was expected due to the inhibitory action of clavulanic acid against β-lactamases [79]. Regarding CNS isolates, none were found to be resistant to neomycin.

One *S. aureus* and one CPS *Staphylococcus* sp. were found to be resistant to oxacillin, while CNS oxacillin resistant isolates belonged to eight species: *S. chromogenes* (5), *S. caprae* (4), *S. lentus* (3), *S. simulans* (3), *S. epidermidis* (2), *S. auricularis* (1), *S. hominis* (1), and *S. warneri* (1). Other authors previously reported the presence of methicillin resistant coagulase-negative staphylococci (MR-CNS) [80,81].

Regarding tetracycline, most *S. aureus* isolates (32/35) were susceptible, while non-susceptible isolates belonged to the following CNS species: *S. caprae* (4), *S. haemolyticus* (3), *S. lentus* (2), *S. capitis* (1), *S. hominis* (2), *S. rostri* (1), and *S. warneri* (1). Tetracycline has been widely used in veterinary medicine, and other studies have reported a higher percentage of resistant isolates: 42.8% [82] and 28.9% [45]. On the contrary, our results show a relatively low percentage of non-susceptible isolates (12.4%). In recent years, there has been an abusive use of more recent antimicrobial molecules, such as cephalosporins and quinolones, that may justify the observed reversal in the patterns of resistance to tetracyclines. To avoid the use of critically important antimicrobials for human medicine, tetracyclines, gentamycin, or cefazolin, a first-generation cephalosporin, may be an option for the control of mastitis in small ruminants. However, there should be a tight control over the development of antimicrobial resistances.

Interestingly, an association between resistance to some antibiotics and animal species was found: penicillin (χ^2^ = 26.931, df = 1, *p* < 0.001), ampicillin (χ^2^ = 26.818, df = 1, *p* < 0.001), oxacillin (χ^2^ = 6.241, df = 1, *p* < 0.05), streptomycin (χ^2^ = 26.231, df = 2, *p* < 0.001), and lincomycin (χ^2^ = 20.831, df = 2, *p* < 0.001). For example, isolates from goats (G) were more resistant than sheep (S) isolates to β-lactams, penicillin (G-43%; S-2%), ampicillin (G-39%; S- = 0%), and oxacillin (G-22%; S-6%). These differences might be due to different management systems, as suggested by Barrero-Domínguez et al. [45], who reported sheep and goat staphylococcal isolates with the same pulsotypes to exhibit distinct resistance patterns.

### 2.6. Antimicrobial Resistance Genes

The 44 biofilm producing isolates were selected for the detection of antimicrobial resistance genes involved in the resistance to β-lactams and tetracyclines, namely, *blaZ*, *mecA*, *mecC*, *tetK*, and *tetM*. Table 2 shows the antimicrobial genes detected in each isolate, along with its antimicrobial resistance profile.

The *bla*Z gene was detected in 15 staphylococcal isolates belonging to the following species: *S. chromogenes* (7), *S. aureus* (3), *S. warneri* (2), *S. auricularis* (1), *S. caprae* (1), and *S. simulans* (1). Unexpectedly, nine penicillin-susceptible isolates harbor the *blaZ* gene, namely *S. chromogenes* (5), *S. warneri* (2), *S. auricularis* (1), and *S. simulans* (1). El Feghaly et al. [83] also reported penicillin-susceptible isolates harboring the *bla*Z gene and concluded that conventional methods for susceptibility testing such as Kirby-Bauer penicillin disk diffusion may not be reliable. According to CLSI [78], there may be rare isolates of staphylococci containing β-lactamase genes, which may result negative in phenotypic β-lactamase detection. Additionally, all isolates resistant to penicillin must be considered resistant to all penicillinase-labile penicillins [78].

No staphylococcal isolates harboring the *mecA* or *mecC* genes were detected, although two isolates were found to be non-susceptible to oxacillin and cloxacillin simultaneously, one only to oxacillin and seven to cloxacillin alone. According to the CLSI (2016), oxacillin disk diffusion testing is not reliable for detecting methicillin resistance, at least in *S. aureus*, and cefoxitin should be used for disk diffusion testing. However, Barrero-Domínguez, Luque, Galán-Relaño, Vega-Pla, Huerta, Román, and Astorga [45] also did not detect the *mecA* gene in a cefoxitin-resistant MRSA strain. Thus, other resistance mechanisms cannot be excluded, namely, overproduction of β-lactamase, modified penicillin-binding proteins, distinct SCCmec elements, as well as putative *mecA* mutations [84,85]. Furthermore, Becker et al. [86] have recently reported the presence of a *mecB* gene in a MRSA strain, negative for both *mecA* and *mecC* genes. However, concerning *mecC* detection in our study, we cannot conclude that the isolates with a negative PCR result did not harbor the *mecC* gene, since no positive control strain was available.

An association was found between the resistance to penicillin (χ^2^ = 11.650, df = 1, *p* < 0.05) and ampicillin (χ^2^ = 15.828, df = 1, *p* < 0.001) and the presence of the antimicrobial resistance gene *blaZ*. The association between resistance to penicillin and ampicillin and the presence of the antimicrobial resistance gene *blaZ* has been reported before by other authors [87,88]. However, no association was detected between the resistance to oxacillin and cloxacillin and the presence of the antimicrobial resistance gene *mecA* for this subgroup of 44 isolates.

Only one *S. aureus* isolate carrying the *tetK* and another one carrying the *tetM* gene were identified. Both showed resistance to tetracycline. A *S. warneri* tetracycline-resistant isolate did not harbor either *tetK* or *tetM* (Table 3). El-Razik, Arafa, Hedia, and Ibrahim [82] found a *S. intermedius* isolate showing intermediate resistance to tetracycline, not harboring *tetK*, *tetL*, *tetM*, and *tetO* genes.

## 3. Materials and Methods

### 3.1. Milk Samples Collection and Bacteriological Analyses

A total of 328 small ruminants (258 goats and 70 sheep), belonging to 23 both traditional and industrial dairy farms in Portugal and Brazil, were used to collect 646 half-udder milk samples (508 from goats and 138 from sheep).

Milk samples were aseptically collected in a sterile bottle after the teat was carefully disinfected with 70% ethanol and the first flush was rejected. The samples were kept refrigerated and transported to the laboratory. Ten microliters of each milk sample were plated onto MacConkey agar (Oxoid, Hampshire, UK, CM0007) and onto blood agar (BA) (Oxoid, Hampshire, UK; CM0271 with 5% sheep blood) and incubated at 37 °C for 24 h to 48 h.

Colonies from BA were transferred to brain heart infusion agar (BHI) (Oxoid, Hampshire, UK, CM1136) and again incubated at 37 °C for 24h for primary identification of the *Staphylococcus* genus through morphological and biochemical characteristics, namely, colony morphology, Gram staining, and catalase reaction, according to Markey et al. [89].

Identification of the species level of all isolates was performed by automated compact system VITEK 2 (bioMérieux, Marcy l’Etoile, France) using GP ID cards following the manufacturer’s instructions. Biochemical identification was confirmed by 16S rRNA gene sequencing whenever necessary, using the primers described previously [90].

### 3.2. Phenotypic Characterisation of Staphylococcal Isolates

#### 3.2.1. Biofilm Detection

Biofilm production was evaluated following the procedures described by Merino et al. [91] with some modifications. In brief, isolates were grown overnight in trypticase soy broth (TSB) at 37 °C. This overnight culture was diluted 1:40 in TSB supplemented with 0.25% glucose, and 200 mL of this cell suspension was used to inoculate microplates. After 24 h of incubation at 37 °C, the microplates were washed three times with 200 μL H_2_O, dried in an inverted position, and stained with 100 μL of 0.25% crystal violet for 2 to 3 min at room temperature. Afterwards, the microplates were rinsed again three times with H_2_O, dried, the dye dissolved in 200 μL ethanol-acetone (80:20), and the absorbance measured at 620 nm. Each assay was performed in triplicate and repeated three times. *Staphylococcus epidermidis* ATCC 12,228 and ATCC 35,984 were used as negative and positive controls, respectively. A blank control was also used.

#### 3.2.2. Antimicrobial Sensitivity Test

The antimicrobial sensitivity test (AST) was performed as described before [77] following the performance standard M02-A11 [92]. Resistance to 16 antimicrobials, belonging to six antimicrobial categories, according to Magiorakos et al. [73], was evaluated: (1) β-lactams-penicillin (*P*), ampicillin (AMP), cloxacillin (OB), amoxicillin + clavulanic acid (AMC), oxacillin (OXA), cephalexin (CL), cefazolin (KZ), ceftriaxone (CRO), cefoperazone (CFP); (2) aminoglycosides-streptomycin (S), gentamycin (CN), neomycin (N); (3) lincosamides-lincomycin (MY); (4) tetracyclines-tetracycline (TET); (5) fluoroquinolones-ciprofloxacin (CIP); and (6) folate pathway inhibitors-cotrimoxazole (sulfamides + trimethoprim) (STX).

For the interpretation of AST results, the CLSI clinical breakpoints M100-S25 were used [78]. Isolates showing intermediate resistance, now called “susceptible increased exposure” [93], were considered non-susceptible. Moreover, isolates resistant to three or more antimicrobial categories were considered multidrug resistant [73].

### 3.3. Molecular Characterisation of Staphylococcal Isolates

The presence of *coa* and *nuc* genes was investigated in all staphylococcal isolates. *nuc*-positive biofilm-producing isolates were selected for the detection of the biofilm production genes, *bap*, *icaA*, and *icaD*, and the antimicrobial resistance genes *blaZ*, *mecA*, *tetK*, and *tetM*. The presence of the *mecC* gene was investigated only for *nuc*-positive biofilm-producing isolates, which were simultaneously resistant to oxacillin and cloxacillin and did not harbor the *mecA* gene.

#### 3.3.1. Rapid DNA Extraction

Total DNA was extracted as described previously [94]. Bacterial cultures were grown for 24 h in BHI (Oxoid, Hampshire, UK, CM1136). After this period, they were transferred to microtubes with 200 μL of ultrapure water and centrifuged at 12,000× *g* for two minutes. Two hundred microliters of sterile saline solution (8.5%) were added to the pellet and centrifuged again at 12,000× *g* for two minutes. Subsequently, 100 μL of 0.05 M NaOH was added to the pellet and boiled for four minutes, then placed immediately on ice. Afterwards, 600 μL of ultrapure water was added to the microtubes and centrifuged at 4000× *g* for three minutes. Subsequently, 400 μL were transferred to a new microtube and stored at −20 °C until use.

#### 3.3.2. PCR Amplification

All amplifications were done in a PTC1148C-MJ Mini thermocycler (BioRad, Hercules, CA, USA).

Amplified DNA fragments were stained with 1X Red Gel (Biotium, Fremont, CA, USA) and run on 1.5% (*w*/*v*) agarose gels with 0.5X TBE (Tris-borate-EDTA) buffer. Different NZYDNA Ladders (NZYtech, Lisbon, Portugal) were used as molecular weight markers, depending on the size of the PCR products.

Agarose gels were photographed under ultraviolet light using the Gel Doc XR+ Gel Documentation System (BioRad Universal Hood II, Philadelphia, PA, USA).

For all PCR amplifications, 50 μL PCR reactions were prepared with 5 μL of DNA template, 1 U GoTaq DNA polymerase (Promega, Madison, WI, USA), 1X Green Go Taq Flexi buffer (Promega, WI, USA), 1.5 mM MgCl_2_ (Promega, WI, USA), 0.2 mM each dNTP (VWR, part of Avantor, Radnor, PA, USA), and 15 pmol each primer (STAB VIDA, Caparica, Portugal). Specific and individual modifications or optimizations were done whenever necessary.

The primers used for amplification of the different genes are listed in Table 3.

For the detection of the *coa* gene, different primer sequences were used. *Staphylococcus aureus* ATCC 25,923 was used as positive control. The first pair of primers, coa-F and coa-R, amplified a 676 bp fragment [55]. The amplification program was as follows: 3 min at 95 °C, and 35 cycles of 30 s at 94 °C, 30 s at 55 °C, 30 s at 72 °C, and finally, 5 min at 72 °C. The second pair of primers, coa2-F and coa2-R, amplified a fragment of 1517 bp [54]. The amplification program comprised an initial denaturation of 45 s at 94 °C, followed by 29 cycles at 94 °C for 20 s, 55 °C for 1 min, and 72 °C for 90 s, and a final extension step of 2 min at 72 °C.

For the amplification of the *nuc* gene, primers nuc-F and nuc-R, amplifying a 267 bp DNA fragment, were used [95]. *S. aureus* ATCC 25,923 was used as positive control and *S. epidermidis* ATCC 12,228 as negative control. The amplification program was the following: 5 min at 94 °C, followed by 37 cycles, consisting of 94 °C for 1 min, 55 °C for 30 s, and 72 °C for 30 s, ending with a final extension step at 72 °C for 7 min.

For detecting the *bap* gene, primers bap-F and bap-R were used for the amplification of a 971 bp fragment [35]. No positive control strain was available. The amplification program was as follows: 94 °C for 2 min, followed by 35 cycles of 94 °C for 45 s, 56.5 °C for 45 s, and 72 °C for 50 s, and finally, 72 °C for 5 min.

Primers icaA-F and icaA-R were used for the amplification of a 1315 bp fragment of the *icaA* gene [96]. *S. epidermidis* ATCC 35,984 was used as positive control. The following amplification program was used: 92 °C for 5 min, followed by 30 cycles of 92 °C for 45 s, 49 °C for 45 s, and 72 °C for 1 min, and a final extension step of 7 min at 72 °C.

For the *icaD* gene, primers icaD-F and icaD-R were used to amplify a 381 bp fragment [96]. *S. epidermidis* ATCC 35,984 was used as positive control. The same amplification program as for *icaA* was used, except for the extension step within the cycles, which was 72 °C for 30 s.

The presence of the *blaZ* gene was detected using primers blaZ-F and blaZ-R, which amplified a 517 bp fragment [97]. *Staphylococcus aureus* ATCC 29,213 was used as positive control and *S. aureus* ATCC 25,923 as negative control [100]. The amplification program was as follows: 94 °C for 4 min, followed by 37 cycles of 94 °C for 1 min, 50.5 °C for 30 s, and 72 °C for 30 s, and finally, 72 °C for 5 min [97].

To detect the *mecA* gene, primers mecA-F and mecA-R were used to amplify a fragment of 532 bp [98]. *Staphylococcus epidermidis* ATCC 35,984 was used as positive control [101] and *S. aureus* ATCC 25,923 as negative control [102]. The following amplification program was used: 94 °C for 2 min, followed by 29 cycles of 94 °C for 30 s, 55 °C for 30 s, and 72 °C for 30 s, and a final extension of 5 min at 72 °C.

Primers mecC-F and mecC-R were used to amplify a 138 bp fragment [99]. No positive control strain was available. The following amplification program was used: 95 °C for 2 min, followed by 30 cycles of 94 °C for 30 s, 50 °C for 30 s, and 72 °C for 30 s, and a final extension of 10 min at 72 °C.

Primers tetK-F and tetK-R were used to amplify a 360 bp fragment of the *tet*K gene [59]. No positive control strain was available. For the amplification of the *tet*M gene, tetM-F and tetM-R were used to amplify a fragment of 158 bp [59]. No positive control strain was available. The amplification program for both *tet* genes was: 94 °C for 2 min, followed by 29 cycles of 94 °C for 30 s, 55 °C for 30 s, and 72 °C for 30 s, with a final step of 5 min at 72 °C.

### 3.4. Data Analysis

The chi-square test of association was used: to assess the relationship between the presence of the *nuc* gene with *Staphylococcus* species; to investigate if the presence of the *nuc* gene was associated with biofilm production; to check if the resistance to antimicrobials was associated with bacterial species and with the animal species from which these were isolated. For the abovementioned analyses, all 137 isolates were considered.

For the subgroup of 44 *nuc*-positive biofilm-producing isolates, the chi-square test of association was performed to evaluate the putative relationship between phenotypic resistance to antimicrobials and the presence of four resistance genes.

Multiple correspondence analysis (MCA) was used as an exploratory data analysis technique to detect a structure in the relationships between bacterial species and resistance to selected antimicrobials, divided either into two (susceptible and resistant) or three classes (susceptible, intermediate, and resistant), depending on the antimicrobial.

All statistical analyses were performed using the software STATISTICA Version 12 (StatSoft, Inc., Tulsa, OK, USA).

## 4. Conclusions

Mastitis aetiology showed to be diverse in the two small ruminant species studied. The most abundant species was *S. caprae*, which, however, was only present in goats.

The *nuc* gene was detected in 67 isolates, of which only 35 were *S. aureus*. Most CNS did not harbor this gene; however, it was detected in more than 50% of *S. warneri*, *S. lentus*, *S. auricularis*, and *S. hyicus*. Although many studies still consider the *nuc* gene as the sole character to identify *S. aureus*, our results have clearly demonstrated that this gene is insufficient, because it is present in numerous staphylococcal isolates other than *S. aureus*.

Most staphylococci were biofilm producers. The *bap* gene was only detected in CNS, while *ica* operon genes were mainly detected in *S. aureus* isolates, suggesting that CNS produce biofilm mainly via Bap, and most *S. aureus* form biofilm through PIA. Furthermore, biofilm-producing staphylococcal isolates not harboring the classical biofilm-production genes *bap*, *icaA*, and *icaD* carry the *nuc* gene. Therefore, the role of the Nuc thermonuclease in staphylococci biofilm formation needs to be further investigated.

Antimicrobial resistance seems to be a growing concern in the treatment of sheep and goat mastitis, with only a low number of isolates (18%) not showing any antimicrobial resistances. Furthermore, CNS were generally more resistant to antimicrobials than CPS. Additionally, an association between animal species and resistance to some antimicrobials was found, suggesting different managing systems for the two species.

All staphylococcal isolates were susceptible to cefazolin and gentamycin. Furthermore, all *S. aureus* isolates were shown to be susceptible to amoxicillin + clavulanic acid and most (32/35) to tetracycline. The use of these antimicrobials to control mastitis may be encouraged to avoid the use of others critically important for human medicine that are currently being used, such as third generation cephalosporins and quinolones. Nevertheless, antimicrobial susceptibility tests cannot be neglected, as the development of resistant strains is always a problem.

Regarding antimicrobial resistance genes, nine penicillin-susceptible isolates exhibited the *blaZ* gene, highlighting the poor reliability of conventional methods for susceptibility testing. Moreover, no staphylococcal isolates harboring the *mecA* or *mecC* genes were detected among those found to be non-susceptible to oxacillin. Hence, other methicillin resistance mechanisms need to be explored.

## Figures and Tables

**Table 1:** *nuc*-positive biofilm-producing staphylococcal isolates and biofilm-associated genes.

Isolate	Origin	Animal	Bacterial Species	*coa*	*nuc*	*bap*	*icaA*	*icaD*
1D	PT	goat	*S. aureus*	+	+	−	+	+
13D1	PT	goat	*S. warneri*	−	+	−	−	−
17D1	PT	goat	*S. aureus*	+	+	−	−	+
44D	PT	goat	*S. aureus*	+	+	−	+	+
47D2	PT	goat	*S. chromogenes*	−	+	+	−	−
50E1	PT	goat	*S. aureus*	+	+	−	+	+
54E1	PT	goat	*S. warneri*	−	+	+	−	−
54E2	PT	goat	*S. warneri*	−	+	−	+	−
55D1	PT	goat	*S. capitis*	−	+	−	−	−
60D2	PT	goat	*S. chromogenes*	−	+	+	−	−
65D	PT	goat	*S. caprae*	−	+	−	−	−
70D	PT	sheep	*S. aureus*	+	+	−	−	+
71E	PT	sheep	*S. aureus*	+	+	−	−	−
72D	PT	sheep	*S. aureus*	+	+	−	−	+
72E	PT	sheep	*S. aureus*	+	+	−	−	+
83D	PT	sheep	*S. aureus*	+	+	−	−	−
B51E	BR	goat	*S. chromogenes*	−	+	−	−	−
B64	BR	goat	*S. chromogenes*	−	+	−	−	−
B76E	BR	goat	*S. chromogenes*	−	+	+	−	−
B101	BR	goat	*S. warneri*	−	+	−	+	−
B159D	BR	goat	*S. chromogenes*	−	+	+	−	−
B159E	BR	goat	*S. chromogenes*	−	+	+	−	−
B190D	BR	goat	*S. auricularis*	−	+	−	−	−
B209D2	BR	goat	*S. simulans*	−	+	+	−	−
B209E	BR	goat	*S. simulans*	−	+	−	−	−
B219D3	BR	sheep	*S. auricularis*	−	+	−	−	−
B219D5	BR	sheep	*S. aureus*	+	+	−	−	−
B223D	BR	sheep	*S. aureus*	+	+	−	−	−
B250D	BR	sheep	*S. auricularis*	−	+	+	−	−
CQ152E1	PT	sheep	*S. aureus*	+	+	−	+	+
CQ185D1	PT	sheep	*S. aureus*	+	+	−	+	+
CQ196E	PT	sheep	*S. aureus*	+	+	−	−	+
CQ201E	PT	sheep	*S. aureus*	+	+	−	−	+
CQ268D1	PT	sheep	*S. aureus*	+	+	−	−	+
CQ270E1	PT	sheep	*S. aureus*	+	+	−	−	−
CQ285D	PT	sheep	*S. aureus*	+	+	−	−	+
CQ286D	PT	sheep	*S. aureus*	+	+	−	−	+
CQ290D1	PT	sheep	*S. aureus*	+	+	−	−	+
CQ290D2	PT	sheep	*S. aureus*	+	+	−	−	+
CQ296D	PT	sheep	*S. aureus*	+	+	−	−	+
CQ335E	PT	sheep	*S. aureus*	+	+	−	−	−
CQ336E2	PT	sheep	*S. aureus*	+	+	−	−	+
CQ349D	PT	sheep	*S. aureus*	+	+	−	−	−
CQ354D	PT	sheep	*S. aureus*	+	+	−	−	+

PT-Portugal; BR-Brazil.

**Table 2:** *nuc*-positive biofilm-producing staphylococcal isolates, phenotypic resistance to selected antimicrobials and their associated antimicrobial resistance genes.

Isolate	Origin	Animal	Bacterial Species	P	AMP	OB	AMC	OXA	TET	*blaZ*	*mecA*	*mecC*	*tetK*	*tetM*
1D	PT	goat	*S. aureus*	R	R	R	S	S	S	+	-		-	-
13D1	PT	goat	*S. warneri*	S	S	S	S	S	S	-	-		-	-
17D1	PT	goat	*S. aureus*	R	R	R	S	S	R	+	-		-	+
44D	PT	goat	*S. aureus*	R	R	S	S	S	S	+	-		-	-
47D2	PT	goat	*S. chromogenes*	R	R	R	R	R	S	+	-	-	-	-
50E1	PT	goat	*S. aureus*	S	S	S	S	S	S	-	-		-	-
54E1	PT	goat	*S. warneri*	S	S	R	S	S	S	+	-		-	-
54E2	PT	goat	*S. warneri*	S	S	R	S	R	S	-	-	-	-	-
55D1	PT	goat	*S. capitis*	S	S	S	S	S	S	-	-		-	-
60D2	PT	goat	*S. chromogenes*	R	R	S	S	S	S	+	-		-	-
65D	PT	goat	*S. caprae*	R	R	S	S	S	S	+	-		-	-
70D	PT	sheep	*S. aureus*	S	S	S	S	S	S	-	-		-	-
71E	PT	sheep	*S. aureus*	S	S	R	S	S	S	-	-		-	-
72D	PT	sheep	*S. aureus*	S	S	S	S	S	S	-	-		-	-
72E	PT	sheep	*S. aureus*	S	S	S	S	S	S	-	-		-	-
83D	PT	sheep	*S. aureus*	S	S	S	S	S	S	-	-		-	-
B51E	BR	goat	*S. chromogenes*	S	S	S	S	S	S	+	-		-	-
B64	BR	goat	*S. chromogenes*	S	S	S	S	S	S	+	-		-	-
B76E	BR	goat	*S. chromogenes*	S	S	S	S	S	S	+	-		-	-
B101	BR	goat	*S. warneri*	S	S	S	S	S	R	+	-		-	-
B159D	BR	goat	*S. chromogenes*	S	S	S	S	S	S	+	-		-	-
B159E	BR	goat	*S. chromogenes*	S	S	S	S	S	S	+	-		-	-
B190D	BR	goat	*S. auricularis*	R	S	S	S	S	S	-	-		-	-
B209D2	BR	goat	*S. simulans*	S	S	S	S	S	S	-	-		-	-
B209E	BR	goat	*S. simulans*	S	S	S	S	S	S	+	-		-	-
B219D3	BR	sheep	*S. auricularis*	S	S	S	S	S	S	-	-		-	-
B219D5	BR	sheep	*S. aureus*	S	S	S	S	S	S	-	-		-	-
B223D	BR	sheep	*S. aureus*	S	S	S	S	R	S	-	-	-	-	-
B250D	BR	sheep	*S. auricularis*	S	S	S	S	S	S	+	-		-	-
CQ152E1	PT	sheep	*S. aureus*	S	S	S	S	S	S	-	-		-	-
CQ185D1	PT	sheep	*S. aureus*	S	S	S	S	S	S	-	-		-	-
CQ196E	PT	sheep	*S. aureus*	S	S	S	S	S	S	-	-		-	-
CQ201E	PT	sheep	*S. aureus*	S	S	S	S	S	S	-	-		-	-
CQ268D1	PT	sheep	*S. aureus*	S	S	R	S	S	S	-	-		-	-
CQ270E1	PT	sheep	*S. aureus*	S	S	R	S	S	S	-	-		-	-
CQ285D	PT	sheep	*S. aureus*	S	S	S	S	S	S	-	-		-	-
CQ286D	PT	sheep	*S. aureus*	S	S	S	S	S	S	-	-		-	-
CQ290D1	PT	sheep	*S. aureus*	S	S	R	S	S	S	-	-		-	-
CQ290D2	PT	sheep	*S. aureus*	S	S	S	S	S	S	-	-		-	-
CQ296D	PT	sheep	*S. aureus*	S	S	S	S	S	R	-	-		+	-
CQ335E	PT	sheep	*S. aureus*	S	S	S	S	S	S	-	-		-	-
CQ336E2	PT	sheep	*S. aureus*	S	S	S	S	S	S	-	-		-	-
CQ349D	PT	sheep	*S. aureus*	S	S	S	S	S	S	-	-		-	-
CQ354D	PT	sheep	*S. aureus*	S	S	S	S	S	S	-	-		-	-

Penicillin (P), ampicillin (AMP), cloxacillin (OB), amoxicillin + clavulanic acid (AMC), oxacillin (OXA), tetracyclines-tetracycline (TET).

**Table 3:** Primer sequences for amplification of the different genes.

Gene	Primer	Sequence	Reference
*coa*	coa-F	5′ ATA GAG ATG CTG GTA CAG G 3′	[55]
	coa-R	5′ GCT TCC GAT TGT TCG ATG C 3′	
*coa*	coa2-F	5′ TA CTC AAC CGA CGA CAC CG 3′	[54]
	coa2-R	5′ GAT TTT GGA TGA AGC GGA TT 3′	
*nuc*	nuc-F	5′ GCG ATT GAT GGT GAT ACG GTT 3′	[95]
	nuc-R	5′ AGC CAA GCC TTG ACG AAC TAA AGC 3′	
*bap*	bap-F	5′ CCC TAT ATC GAA GGT GTA GAA TTG CAC 3′	[35]
	bap-R	5′ GCT GTT GAA GTT AAT ACT GTA CCT GC 3′	
*icaA*	icaA-F	5′ CCT AAC TAA CGA AAG GTA G 3′	[96]
	icaA-R	5′ AAG ATA TAG CGA TAA GTG C 3′	
*icaD*	icaD-F	5′ AAA CGT AAG AGA GGT GG 3′	[96]
	icaD-R	5′ GGC AAT ATG ATC AAG ATA C 3′	
*blaZ*	blaZ-F	5′ AAG AGA TTT GCC TAT GCT TC 3′	[97]
	blaZ-R	5′ GCT TGA CCA CTT TTA TCA GC 3′	
*mecA*	mecA-F	5′ AAA ATC GAT GGT AAA GGT TGG C 3′	[98]
	mecA-R	5′ AGT TCT GCA GTA CCG GAT TTG C 3′	
*mecC*	mecC-F	5′ GAA AAA AAG GCT TAG AAC GCC TC 3′	[99]
	mecC-R	5′ GAA GAT CTT TTC CGT TTT CAG C 3′	
*tetK*	tetK-F	5′ GTA GCG ACA ATA GGT AAT AGT 3′	[59]
	tetK-R	5′ TAG TGA CAA TAA ACC TCC TA 3′	
*tetM*	tetM-F	5′ AGT GGA GCG ATT ACA GAA 3′	[59]
	tetM-R	5′ CAT ATG TCC TGG CGT GTC TA 3′	
